# The Impact of Positive Emotional Appeals on the Green Purchase Behavior

**DOI:** 10.3389/fpsyg.2022.716027

**Published:** 2022-06-02

**Authors:** Jianming Wang, Xincheng Yang, Zhengxia He, Jianguo Wang, Jing Bao, Jian Gao

**Affiliations:** ^1^School of Business Administration, Zhejiang University of Finance and Economics, Hangzhou, China; ^2^School of Economics, Hangzhou Normal University, Hangzhou, China

**Keywords:** positive emotional appeal, field experiment, green purchase behavior, emotional arousal, middle doctrine

## Abstract

The practice of green consumption has become a common initiative of the international community. Existing studies have shown that emotional factors have an important impact on consumer behavior, but few scholars in the field of green consumption behavior have explored the relationship between positive emotions and green purchase behavior in specific dimensions. This study creatively put forward two dimensions and four kinds of positive emotional appeals which include the cherishing appeal, the yearning appeal, the proud appeal, and the admiring appeal. Through the conceptual model analysis based on the data, the effects of different four positive emotional appeals on consumers’ perceived green value, perceived green responsibility, and green purchase behaviors were confirmed. The results indicated that the four kinds of positive emotional appeals, perceived green value, perceived green responsibility, and green purchase behavior were positively correlated. Perceived green responsibility and perceived green value have significant mediating effect on the relationship between positive emotional appeals and green purchase behaviors. Middle doctrine significantly moderates the relationship between positive emotional appeals (Cherishing VS. Yearning) and perceived green value.

## Introduction

Nowadays, the global natural ecological environment is deteriorating day by day, and a series of pollution problems emerge one after another. Therefore, it is imperative to attach importance to and actively advocate green consumption behavior. The 19th national congress of the communist party of China (CPC) once again stressed the necessity to establish and practice the concept that clear waters and green mountains are valuable assets. However, in general, residents’ green purchase intention and behaviors are not popular, and people are not willing to pay a premium for the green properties of products. The government and society have made great efforts to promote environmental protection and green consumption, but the effect is not satisfactory. The report of the International Institute of Green Finance of the Central University of Finance and Economics pointed out that the green penetration rate of consumers of different age groups in China is quite different and the environmental protection concept is quite different among different age groups. Consumers of some age groups do not yet have a good awareness of green consumption.^1^ The reason is that propaganda is mostly spread in the form of preaching, and the content is dull and unappealing. So, it is difficult to arouse consumers’ green purchase desire and change their original purchase concept. Kollmuss and Agyeman (2002) and Tanner and Kast (2003) found that rational cognition had a limited effect on the factors influencing green purchase behavior. Emotional factors had a significantly higher effect on green consumption than cognitive factors (Kanchanapibul et al., 2014). Therefore, more and more scholars gradually realized emotion plays an important role in green purchase behavior. More research breakthroughs were transferred to a relatively irrational variable-emotion (Peattie, 2010). Past research proved the relationship between emotion and green purchasing behavior to some extent (Meneses 2010; Onwezen et al., 2013, 2014; Antonetti and Maklan, 2014; Wang, 2015; Wang et al., 2017). However, a series of practical problems remains, such as how emotional factors influence in consumers’ green purchase behavior, which factors can mediate or moderate on this path, and which kind of emotions can significantly promote green purchase behavior, scholars have not come with sufficient conclusions. Wang et al. (2017) pointed out that from the perspective of emotional objects, positive emotions include not only the emotion toward a good objective environment (such as love for the environment), but also the emotion toward proper human behaviors (such as pride or admiring for the proper behaviors). In our view, the positive emotional dimension for a better objective environment, “yearning” is people’s expectation of better things in the future, which makes people full of hope and makes continuous efforts for them. There is no doubt that people yearn for more comfortable living conditions, a more beautiful environment, and a better life. According to the report to the 19th national congress of CPC, we should “always take the people’s aspiration for a better life as our goal.” Therefore, it is of great practical value to deeply explore the positive emotion of “yearning.” “Cherishing” is a kind of emotion opposite to “yearning,” which is the satisfaction and cherishing of the beautiful things we have at present. Both of these two emotions belong to the positive emotional dimension aiming at the beautiful objective environment. In order to solve the above problems more effectively, this study creatively put forward two specific dimensions of four kinds of positive emotional appeals and make in-depth exploration. One of the dimensions is the emotion toward a better environment, including the cherishing appeal and the yearning appeal. The other dimension is the emotion toward the appropriate individual environmental behavior, including the proud appeal and the admiring appeal. According to different dimensions, this study conducted a thorough research by group on the influence path and effect of positive emotional appeals on green purchase behavior, providing theoretical support for effectively promoting consumers to practice green purchase behavior.

## Literature Review and Hypotheses Development

### Emotional Appeal

Rajesh et al. (2001) believe that advertising appeal is a strategic way to display prominent features of products. In the existing research, many scholars have done a lot of research on the marketing effects of different green appeal advertisements or consumers’ reactions to different green appeal advertisements from different perspectives (Schuhwerk and Lefkoff-Hagius, 1995). The analysis dimension includes green and non-green appeals (Ku et al., 2012), environmental interests and economic interests appeals (Xu et al., 2015), self-interest and others’ interests appeals (Zhi et al., 2017), long-term interests and short-term interests appeals (Xu et al., 2015), abstract and specific appeals (Yang et al., 2015), mandatory and suggested appeals (Kronrod et al., 2012), strong appeals and non-strong appeals (Chen and Deng, 2017), real environmental demands and false environmental demands (Lei and Kunhao, 2016), etc. However, researchers have paid less attention to emotional appeals. Existing research on emotional appeal mainly focuses on the difference in the impact of rational appeal and emotional appeal (Matthes et al., 2014) and there is a relatively lack of research specifically on emotional advertising appeal. Compared with rational appeal, emotional appeal has more strong emotional elements, which can stimulate consumers’ purchase desire and generate psychological identification. Chan (1996) and other scholars argued that compared with emotional appeals, rational appeals advertisements lack vividness and the content is boring, which is difficult to capture people’s interest. Therefore, it is very important and necessary to pay attention to the research on emotional appeal.

### Environmental Positive Emotion

According to the research results of scholars, the influence of different emotions on consumers’ purchase behavior is quite different. Scholars often use the emotion dichotomy, which divides emotion into negative and positive dimensions according to the experience structure (Watson and Tellegen, 1985; Watson et al., 1999). Positive emotions are a series of positive feelings, including enthusiasm, gratitude, excitement, happiness, and pride (Qiu et al., 2008). Meneses (2010) conducted a special study on emotion, and the results showed that positive emotion could increase consumers’ intrinsic motivation to purchase products, and the driving effect of positive emotional factors is significantly greater than negative emotional factors. For consumers, positive emotions are very important. However, current research lacks a discussion of the differences in the effects of different types of positive emotion. In the field of green marketing research, the difference in the impact of different types of positive emotions on consumers and the formation mechanism of this difference should be paid attention to by researchers.

Wang (2015) adopted grounded theory approach and sorted out the raw materials of the in-depth interviews. He found environmental emotion has dual factors (both positive and negative) and six dimensions, in which positive environmental emotions including the feeling of loving the environment, admiring, and being proud of the green behavior. The category of the feeling of loving the environment contained a series of initial concept and the two most typical and important initial concepts in this category are cherishing and yearning. Based on the qualitative result of Wang (2015), we derive that positive environmental emotion contained four dimensions, namely, cherishing, yearning, proud, and admiring.

#### Cherishing

Cherish refers to the psychological activity of consciously cherishing the objective things, others, and themselves. The awareness of cherishing is relatively stable after it is formed in an individual and will not fade or even disappear due to changes in the environment. It will not go away, but it will change. Once the cherishing consciousness is formed, it will continue to strengthen under the influence of positive conditions; on the contrary, it will gradually weaken under the influence of negative conditions. Shen et al. (2011) constructed the quality scale of college students’ gratitude from the three dimensions of cognition, emotion, and behavior, in which the emotional dimension included three sub-dimensions such as sense of cherishing.

#### Yearning

Yearning refers to the desire to get or to achieve because of love or envy of a certain thing or state, and it is the “expected prospect” of people. Yearning is accompanied by people’s good wishes and positive goals, which can inspire people to be full of motivation to move forward and can make people focus on the goals they want to achieve and focus on those goals. Yearning can motivate people to develop in a good direction. It will form a beautiful picture in people’s imagination, and it will keep appearing in their minds. People can form the hint of continuous self-motivation by imagining the achievement of goals, motivating individuals to make continuous efforts toward the vision of their dreams. That is, yearning helps arouse people’s imaginations and expectations, which motivate individuals to take action to achieve their goals. When yearning enters the expectation into the subconscious, this kind of vision for the future will become the unswerving “belief” of the individual. Under the guidance of the belief, the individual will work hard toward the desired prospect. People who are full of yearning focus on the future and hope to achieve the desired state through present and future efforts.

#### Proud

Proud is a positive emotion that includes a sense of accomplishment and self-satisfaction (Antonetti and Maklan, 2014) and is also a self-conscious emotion (Feng and Zhang, 2007). Williams and DeSteno (2009) pointed out that the emotion of proud is an individual’s self-satisfaction through his own efforts. In real life and existing research, proud is often associated with positive words such as “sense of achievement” and “confidence,” which reflect an individual’s social emotions and achievement orientation. Many studies have confirmed that pride has a positive impact on green purchasing behavior (Onwezen et al., 2013, 2014; Antonetti and Maklan, 2014). Harth et al. (2013) found that proud is the strongest predictor of environmental protection behaviors. Wang and Wu (2015) used an experimental method to explore the impact of four green buying emotions of proud, appreciation, guilt, and contempt on green buying behavior. They believed that proud is the best factor for cultivating consumers’ awareness of green consumption.

#### Admiring

Admiring means approval and appreciation, which refers to an individual’s positive emotional attitude toward others and this emotion also promotes oneself (Keltner and Haidt, 1999; Algoe Sara and Jonathan, 2009). It is a positive emotion (Sarapin et al., 2015) that has lesser degree than “awe” (Zagzebski, 2015). Social cognitive theory holds that people tend to be more inclined to learn from people they admire (Matsueda and Akers, 1999; Bandura, 2001; Wareham et al., 2009). Therefore, admiring is usually paid attention to by researchers because individuals can learn a certain ability through this emotional stimulation. In an empirical study, Algoe Sara and Jonathan (2009) pointed out that an individual’s admiring of others leads to better performance in related aspects. Helen et al. (2010) pointed out that individual learning can be improved through admiring. However, in the field of green purchasing, scholars have paid little attention to the role of admiring in shaping individual behavior.

#### Summary

In the field of green marketing research, there are two gaps in current research: (i) lack of research specifically on emotional appeal; (ii) lack of research on the impact of different dimensions of positive emotional appeal on consumers’ green consumption behavior. Investigating the role of positive emotions in different dimensions can help companies conduct green marketing more effectively. Therefore, this paper focuses on the differences and effects of the four representative dimensions of positive emotional appeals on consumers’ green purchasing behavior (These four dimensions can be divided into two groups: emotion for better environment and emotion for appropriate personal environmental behavior). The former includes the cherishing and yearning appeal, which, respectively, represent the appreciation of the present beautiful environment and the expectation of the future beautiful environment. The latter includes the proud and admiring appeal, which, respectively, represent the pride in one’s own appropriate environmental behavior and the appreciation of others’ appropriate environmental behaviors.

### Theoretical Background

#### The Broaden-and-Build Theory of Positive Emotions

Fredrickson (1998) proposed broaden-and-build model of positive emotion theory and demonstrated the specific functions of positive emotion, which believes that positive emotion can affect individual’s psychological state, triggering individual’s pleasure, and then changing his thought. Positive emotion produces an action activation—a tendency to avoidance or proximity. Fredrickson (1998) believed that positive affect is not only associated with a general tendency to activate activities but also associated with a specific tendency to act. Positive affect can promote the continuity of behavior development. Under the influence of positive emotions, individual will issue more positive instructions and the cognitive level will be accordingly improved, thus remarkably enhancing the possibility of the corresponding behavioral tendency. Stimulated by positive emotional appeals, the brains will naturally make more positive reactions. In addition, positive emotions have a positive effect on an individual’s thoughts and actions. Based on this theory, it is not only consumers’ positive emotion which has an expansive influence on their thinking and specific behavioral tendency—green purchase behavior, but also has a positive and long-term impact on their future cognition and behavior. The Broaden-and-Build Theory of Positive Emotions is the basis of this study, which shows that the appeal of positive emotion can indeed promote green consumption behavior.

#### Prospect Theory

**Figure 1:**
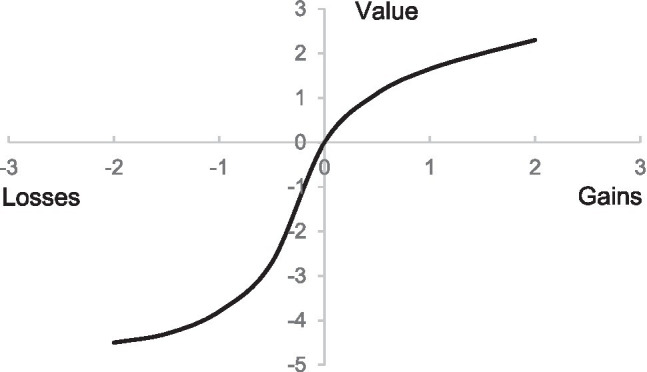
Value function of prospect theory.

Tversky (1979) put forward prospect theory from the perspective of cognitive psychology on the basis of the deficiencies of expected utility theory in explaining individual behavioral decision making. The theory points out that when decision makers make future risk choices, they use the value function to evaluate the value. The measurement of the value function is mainly based on the degree of deviation from the reference point as shown in Figure 1. The gains and losses perceived by individuals are relative to the reference point. The valuation of these gains and losses varies systematically based on the slope of Prospect Theory’s value function, which is steeper closer to the reference point (i.e., diminishing sensitivity) and steeper on the loss side than on the gain side of the reference point (i.e., loss aversion, Tversky, 1979). Differences between outcomes along a steeper part of the value function have a greater influence on subsequent decisions (Larrick et al., 2009; Ren, 2015; Wallace and Jordan, 2017).

We believe that consumers will choose different reference points when stimulated by different types of emotional appeals. Yearning appeals make consumers focus on the future and therefore use the future situation as a reference point. The cherishing appeal emphasizes the protection of the existing environment, so the current state is set as a reference point. Admiring appeal will make consumers pay more attention to the green consumption behavior of others, that is, they will set the green consumption behavior of others as the reference point. On the contrary, proud appeal will make consumers pay more attention to themselves, so they tend to set their own behavior as a reference point. Due to the difference in the selection of reference points, it will further affect the perception of consumers. Therefore, based on the above discussion, this article will use prospect theory to explain consumer perception differences.

### The Direct Effects and Mediating Effects

This paper will first discuss the difference in the effects of cherishing appeal and yearning appeal on consumers’ green purchasing behavior and the mechanism, both of which belong to the dimension of emotions toward a better environment. We suggest that yearning appeal has a greater positive impact on consumers’ green consumption behavior than cherishing appeal and the relationship between the effects is mediated by perceived green value. There are several reasons as follows:

Firstly, under different positive emotional appeals, consumers make different responses. According to the broaden-and-build theory of positive emotions, positive emotions have the role in expanding people’s thoughts and actions, having a relatively lasting influence on individuals (Liu, 2015). The expansion role is positively correlated with the intensity of positive emotions, that is, the stronger the positive emotions are, the corresponding greater expansion role on the individual’s thought and actions. For instance, the empirical research of Chan and Lau (2000) shows that ecological consumption sentiment has a significant positive impact on ecological consumption behavior. The stronger the individual’s emotion toward ecology and resources, the stronger the tendency to implement green consumption behavior. The sense of cherishing is a positive emotion toward the present beautiful environment, while the sense of yearning is a positive emotion toward the future better environment. It is common sense that yearning appeals bring more expectations and motivation than cherishing, resulting in a stronger motivation to engage in green consumption.

Secondly, both the cherishing appeal and the yearning appeal reflect the concern for the environment. This will lead consumers to associate the purchase of green products with protecting the environment. Consumers will consider the value of purchasing green products to the environment, that is, pay attention to the green value of products. This paper will use reference points to explain the difference in the impact of cherishing appeal and yearning appeals on consumers. Yearning appeal will make consumers pay more attention to the beautiful ecological environment in the future, which will set the beautiful ecological environment in the future as a reference point. After purchasing green products or conducting green consumption, will bring the existing environmental conditions closer to the desired environmental conditions. According to prospect theory, a “reference point” divides the space of outcomes into regions of gain and loss (Tversky, 1979). Outcomes above the reference point are evaluated as gains and outcomes below the reference point are evaluated as losses. In the value function, consumers set the future beautiful ecological environment as a reference point. The current ecological environment status is on the left side of the reference point, which means “losses.” If consumers do not engage in green consumption, the gap between the current situation and the future situation cannot be narrowed, which means consumers cannot reduce their losses. According to the loss aversion effect, individuals are more reluctant to accept “losses” than “gains” when faced with the same size loss and gain. In order to avoid losses, individuals will increase the motivation to engage in a certain behavior. Every time a consumer buys a unit of a green product, it means approaching the reference point, that is, reducing losses. At this time, the green value brought by the green product is positive. On the contrary, the cherishing appeal will make consumers pay more attention to the existing ecological environment and consumers will take the current ecological environment as a reference point. At this time, purchasing products that are harmful to the environment means destroying the existing ecological environment and moving the environmental conditions away from the reference point (i.e., existing ecological environment). However, buying green products is only seen as a means to maintain the current state. Therefore, compared with yearning appeal, cherishing appeal leads to less green value of products perceived by consumers.

In addition, it is worth noting that the previous behavior of consumers determines the current ecological environment. Under the condition of cherishing appeal, no matter whether consumers are satisfied or dissatisfied with the current ecological environment, green purchasing behavior can only maintain the status quo. Therefore, cherishing appeals brings less perceived green value.

Thirdly, perceived green value refers to individual’s subjective evaluation for the effectiveness of green products or green consumption. Liu et al. (2020) found that perceived green value plays an intermediary role between the target framework and green consumption intention. Scholars pointed out that consumers’ perceived green value is significantly correlated with individuals’ ecological attitudes and green consumption behaviors (Berger and Corbin, 1992). For example, consumers believe that their green purchase behavior has a positive effect on the improvement of the ecological environment, which represents the perceived green value is strong. At that time, consumers attempt to practice more green purchase behaviors conducive to environmental protection, which plays a further strengthening role on the perceived green value. Besides, Hines et al. (1987) argued that the level of consumers’ perceived green value would affect the degree to which they adopted pro-environmental behaviors. In addition, the results of Maloney et al. (2014) indicated that consumers’ perceived value was positively correlated with green purchase behavior.

In summary, compared with cherishing appeals, yearning appeals have a more positive effects on consumers’ green purchasing behavior. This is mediated by perceived green values. Yearning appeals can lead to higher perceived green values, due to the choice of reference points and the intensity of emotion. Perceived green value can have a significant effect on green purchase behavior. Cherishing appeals and yearning appeals may also make consumers consider their own green responsibilities. Cherish the appeal makes consumers pay attention to their responsibility to maintain the existing environment and yearning for the appeal makes consumers pay attention to their responsibility to improve the environment. Although the two are different, we believe that the difference in intensity is not significant. Therefore, we suggest the relationship between emotions toward a better environment appeals (i.e., cherishing appeal and yearning appeal) and green purchase behavior is not mediated by consumers’ perceived green responsibility.

Hence, we propose the following:

*H1:* Compared with the cherishing appeal, yearning appeal has more positive effects on the consumers’ green purchase behavior.

*H2:* The relationship between emotions toward a better environment appeals (i.e., cherishing appeal and yearning appeal) green purchase behavior is significantly mediated by consumers’ perceived green value.

Next, this paper will discuss the differences in the effects of proud appeal and admiring appeal on consumers’ green purchasing behaviors and their mechanisms, both of which belong to the dimension of emotion toward the appropriate individual environmental behavior. We suggest that compared with proud appeal, admiring appeal has a greater positive effect on consumers’ green consumption behavior, and the relationship between them is mediated by perceived green responsibility. The reasons are as follows:

Firstly, as far as the positive emotions for the individual’s environmentally appropriate behavior are concerned, compared with self-recognition or self-satisfaction, recognition, and appreciation from others are more effective in motivating individuals to practice proper behaviors. The view of social cognitive theory is that people tend to be more inclined to learn from people or their behaviors they admire (Matsueda and Akers, 1999; Bandura, 2001; Wareham et al., 2009; Han et al., 2017). People will feel affirmation and appreciation for the actions of others to protect the environment. Furthermore, individuals can learn certain abilities and perform better through this emotional stimulation. People often voluntarily learn from people or behaviors they admire sincerely. Compared with the recognition of their own environmental protection behaviors, the individual’s appreciation of others’ contribution to the improvement of the environment has more stimulus for them to follow their example and practice the same green purchase behavior and consumers will be aware of the good green purchasing behaviors of others in society. Therefore, compared with the proud appeal, the admiring appeal has a stronger positive impact on consumers’ green product purchase behavior. Furthermore, both proud appeal and admiring appeal are related to individuals, so consumers will consider aspects related to their own responsibilities when stimulated by one of these two appeals. Although both proud and admiring appeal bring positive psychological experience to consumers, it is admitted that comparing with the proud appeal, the admiring appeal will cause consumers to compare themselves with other individuals and further improve their perception of their own green responsibility. Therefore, we suggest that admiring appeal generates stronger perceived green responsibility than proud appeal.

Secondly, we also use reference points to explain the differences in the impact of proud appeal and admiring appeal on consumers’ green buying behavior. Admiring appeal will make consumers pay more attention to the green consumption behavior of others, that is, they will set the green consumption behavior of others as the reference point and compare their own purchasing behavior with the green purchasing behavior of others. According to prospect theory, outcomes above the reference point are evaluated as gains and outcomes below the reference point are evaluated as losses. When the behavior of others is set as the reference point, the individual’s own behavior is on the left side of the reference point and purchasing green products means reducing “losses.” In other words, close the gap with others by purchasing green products. On the contrary, proud appeal will make consumers pay more attention to themselves, that is, set their own behavior as a reference point, and the result of further green purchasing behavior at this time means “gains.” In other words, buying green products means improving oneself and gaining more sense of achievement. According to the loss aversion effect, individuals are more reluctant to accept “losses” than “gains.” Therefore, compared with the proud appeal, the admiring appeal has a stronger positive impact on consumers’ green product purchase behavior. Besides, for the same reason as the first point, under the stimulation of admiring appeals, consumers will be aware of the good green purchasing behaviors of others in society, which further enhances consumers’ perceived green responsibility.

Thirdly, Lao (2013) defined green consumption as a responsible and sustainable consumption behavior that individuals make efforts to protect the natural environment so as to minimize the negative impact on the environment in the consumption process. As a crucial internal driving factor, environmental responsibility somehow determines the way people purchase behaves. In face of green products, for example, consumers with a higher sense of environmental responsibility tend to have a higher purchase intention and be more actively in implementing green purchasing behaviors. Hines et al. (1987) proposed responsible environmental behavior model, which demonstrates a strong correlation between the individual’s sense of responsibility and the environmental behavior. Compared with the individuals without the sense of environmental responsibility, the individuals with the sense of environmental responsibility usually showed more environmentally responsible behaviors, because it indirectly affected the individual’s environmental behavior. Responsibility not only affects consumers’ green purchase behavior, but also mediates the relationship between consumers’ psychological factors and purchase behavior. Wang and Zheng (2011), in the process of exploring the path of psychological awareness factors to consumers’ ecological civilization behavior, found that sense of responsibility can be used as an mediating variable between psychological factors and ecological civilization behavior. The result showed that resource and environment emotions influence consumers’ sense of green responsibility, thus affecting individuals’ ecological civilization behavior.

In summary, compared with the proud appeal, the admiring appeal has a more positive impact on the green purchasing behavior of consumers. This is mediated by perceived green responsibility. Admiring appeals can lead to higher perceived green responsibility and perceived green responsibility can have a significant impact on green purchasing behavior. Moreover, stimulated by proud appeals or admiring appeals, consumers pay more attention to the sense of achievement brought by purchasing green products or narrowing the gap with others rather than the green value of green products. Therefore, we suggest that the relationship between emotions toward the appropriate individual environmental behavior appeals (i.e., proud appeal and admiring appeal) and green purchase behavior is not mediated by consumers’ perceived green value. Hence, we propose the following:

*H3:* Compared with the proud appeal, admiring appeal has more positive effects on the consumers’ green purchase behavior.

*H4:* The relationship between emotions toward the appropriate individual environmental behavior appeals (i.e., proud appeal and admiring appeal) and green purchase behavior is significantly mediated by consumers’ perceived green responsibility.

### Moderating Effects

Consumers’ psychological factors and situational factors exert a great impact on the green purchase behavior. Among multiple situational factors, individual values occupy an important position. Thøgersen and Ölander (2002), for example, found that values are the distal determinants of behavior, and they need to function through adjacent variables, such as perceived behavioral effectiveness. It is common sense that China has a long history of traditional culture. Values such as Taoism, Buddhism, and Confucianism have a subtle influence on individuals’ behaviors, shaping their unique ways of thinking and decision making. Confucianism, the most representative of values in China, has the most profound and extensive influence (Pan et al., 2009). The middle doctrine is the core of Confucianism, embodying all Confucian values and virtues. It is not only a principle of dealing with affairs, but also a dialectical way of thinking that rejects extreme and advocates timely adaptation (Yang, 2009). The middle doctrine is an important manifestation of Chinese traditional cultural values and a crucial component of our national social psychology (Du et al., 2014), which make a far-reaching consequence on individual cognition and behavior. For individuals who have a higher level of middle doctrine, different emotional appeals have little effect on their psychology and behavior. The reason is the individuals who have a higher level of middle doctrine are likely to pay more attention to the harmony between man and nature, and viewing problems as a whole so as to pursue the balance of personal interests and social interests. Therefore, they are less affected by emotional appeals. On the contrary, individuals with a lower level of middle doctrine tend to pay more attention to specific and short-term impacts, so the impact of different emotional appeals on them is more pronounced. In other words, middle doctrine negatively moderates the impact of emotional appeals. Individuals with higher middle doctrine values are less susceptible to emotional appeals. Hence, we propose the following:

*H5a:* The middle doctrine negatively moderates the influence of positive emotional appeals (cherishing vs. yearning) on perceived green value.

*H5b:* The middle doctrine negatively moderates the influence of positive emotional appeals (cherishing vs. yearning) on green purchase behavior.

*H5c:* The middle doctrine negatively moderates the influence of positive emotional appeals (proud vs. admiring) on perceived green responsibility.

*H5d:* The middle doctrine negatively moderates the influence of positive emotional appeals (proud vs. admiring) on green purchase behavior.

Based on the broaden-and-build model of positive emotion theory and prospect theory, this study deeply explores the relationship and mechanism between the positive emotional appeals and green purchase behavior, focusing on comparing differences in stimuli effects of different types of positive emotions, at the same time investigating the moderating role of middle doctrine.

**Figure 2:**
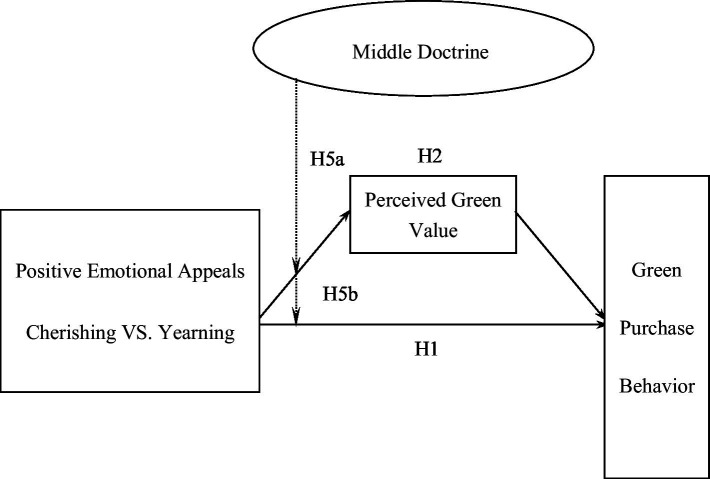
Conceptual model of this study (dimensions of emotions toward a better environment appeals).

**Figure 3:**
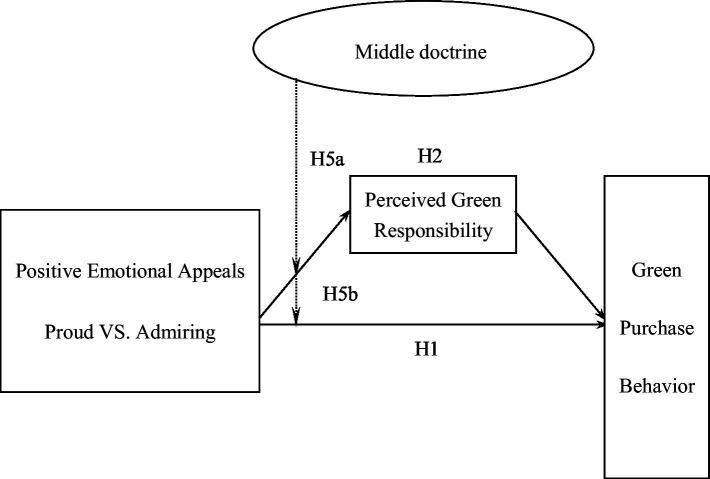
Conceptual model of this study (dimensions of emotions toward the appropriate individual environmental behavior appeals).

The conceptual model of this study is shown in Figures 2, 3. Since emotional appeals of different dimensions are mediated by different mediating variables, there are two model diagrams in this study, in which the solid line represents the influence effect and the dotted line represents the moderating effect. Positive emotional appeal is the explanatory variable, perceived green value and perceived green responsibility are mediating variables, middle doctrine is the moderating variable, and green purchase behavior is outcome variable.

## Materials and Methods

### The Experiment Design

Energy-saving refrigerators and air conditioners are often used as products in previous green consumption experiment because of their green environmental properties. After searching in various ways, this study chose a relatively novel green product—green power certificate, which can fully reflect the consumer’s sense of responsibility, as the experimental product. The green certificate is an electronic certificate for the green electricity produced by power generation enterprises with a unique code identification. Governments, enterprises, and individuals all can subscribe for green certificate on the information platform. On the one hand, the green certificate trading system can guide and regulate the priority consumption of renewable energy power, effectively improve the consumption of green power, reduce the speed of thermal power generation, and promote the efficient use of renewable energy, which is of great significance to the governance of environmental pollution. On the other hand, China’s renewable energy subsidy cumulative gap is large and unsustainable, so the introduction of green certificate system can establish a long-term mechanism for the development of renewable energy. To sum up, now that subscribing the green certificate is a kind of green purchase behavior, regarding the green certificate as an experimental product in this study is scientific and feasible.

In this study, four self-made videos (picture + text + voice + background music) were used as stimulation materials, which had a stronger sense of substitution than simple text description and enabled the respondents to have a deeper understanding of the experimental materials in a more vivid and accurate form. Each video has a short paragraph of text in front of the simple and popular introduction of green certificate, and then follow closely to reflect the corresponding feelings of green video.

By reviewing the existing relevant literature and drawing on the research experience of relevant scholars, this paper summarizes the measurement items of consumers’ perceived green value, perceived green responsibility, green purchase behavior, emotional arousal, and middle doctrine in this study. Likert seven-point scale was used to measure all the items in the questionnaire. The specific measurement items of each variable are shown in Table 1.

**Table 1:** Measurement items of each variable.

Measured variables	Measurement item	Resource
Perceived green value	Subscribing to green electricity certificate helps to save fossil energy and improve energy structure. Subscribing to green electricity certificate helps to cut carbon emissions and mitigate climate change. Subscribing to the green electricity certificate helps to reduce air pollution and improve air quality. Subscribing to the green electricity certificate helps to promote clean energy and sustainable development in China.	Venkatesh et al., 2003
Perceived green responsibility	I have the responsibility to do my best to save resources and protect the environment. No matter what others do, I will save resources and protect the environment. Although my personal power is very small, I also want to contribute to environmental protection. In order to save resources and protect the environment, we should try our best to use clean energy.	Abrahamse and Steg, 2009
Green purchase behavior	I would like to collect and learn more about subscribing to the green electricity certificate. I am willing to subscribe to the green electricity certificate. I would like to recommend my friends and relatives to know and subscribe to the green electricity certificate. This video will prompt me to subscribe to the green electricity certificate.	Dodds et al., 1991; Darley and Smith, 1993
The middle doctrine	Everything should be kept in line and I do not go to extremes. Harmony is the most important thing. Try not to conflict with others. When you make achievements, you should try to remain humble and low-key. When you disagree, you should find a compromise acceptable to all.	Pan et al., 2014

### Sample Description

In this study, the positive emotional appeal videos and the measurement questionnaire were edited into four online links and randomly distributed to the respondents through the Internet. The experiment was conducted through respondents watching the video and filling the corresponding questionnaire, the period of which is August to September 2018. After eliminating the invalid questionnaires, a total of 433 valid questionnaires were collected in this experiment and the sample effective rate was 89.2%. In these 433 valid questionnaires, 48.3% were male, and 51.7% were female. Age distribution is generally below 44 years old, accounting for 97.2% of the total sample size. Education is mainly concentrated in the undergraduate degree and above, because they were more capable of understanding the study and more willing to participate in the experiments.

### Controllability Test

First, we test the manipulation of four emotional awakening videos. The respondents watched four emotional awakening videos, and then, they fill in the first four questions of the questionnaire. We test the validity of video by the scores of the items (7 = totally agree; 1 = totally disagree). The items include as: (1) After watching video, I will cherish the beautiful ecological environment more. (2) After watching video, I will be more yearning for a better ecological environment in the future. (3) If I subscribe to the green power certificate, I will be proud of my behavior. (4) If someone else subscribes to the green power certificate, I will admire his behavior.

Single factor analysis of variance by 433 samples data, we found that the average score of the cherishing appeal in item (1) was significantly higher than that of other emotions, the average score of the yearning appeal in item (2) was significantly higher than that of other emotions, the average score of the proud appeal in item (3) was significantly higher than that of other emotions, the average score of the admiring appeal mean in item (4) was significantly higher than that of other emotions. This indicates that the experiment of different positive emotional appeals in this study was successfully manipulated.

### Reliability and Validity Test

#### Reliability Test

In this paper, the internal reliability index was used to measure the reliability of the scale. As shown in Table 2, Cronbach’s alpha value of all variable items was greater than 0.9, indicating the consistency and stability of the measurement questionnaire.

**Table 2:** Reliability and validity test.

Measured variables	1	2	3	4
1 Perceived green value	0.952			
2 Perceived green responsibility	0.676	0.877		
3 Green purchase behavior	0.778	0.684	0.938	
4 The middle doctrine	0.713	0.651	0.648	0.880
Cronbach’s alpha	0.974	0.929	0.965	0.932
AVE	0.908	0.770	0.879	0.775
CR	0.975	0.931	0.967	0.932

*The diagonal value is the square root of AVE and the lower left part is the Pearson correlation coefficient between latent variables*.

#### Confirmatory Factor Analysis

Confirmatory factor analysis was performed using AMOS 24.0 and the results were as follows: absolute fit indices $\chi^{2}/df$ =2.954 < 3, RMSEA = 0.061 < 0.08, GFI = 0.926, AGFI = 0.898; incremental fit measurement NFI = 0.968, TLI = 0.973, CFI = 0.978. The above results show that the model fits well.

#### Convergent Validity

The output of AMOS24.0 shows that the standardized factor loadings of each measurement item on its corresponding latent variable are between 0.89 and 0.97. As shown in label 2 the average variance extracted (AVE) of each latent variable was greater than 0.5 and the composite reliability (CR) was greater than 0.8. The above results indicated that the scale of this study has good convergent validity.

#### Discriminant Validity

As shown in Table 2, the square root of the AVE value of each latent variable is greater than the correlation coefficient between the latent variable and other latent variables, indicating that the discriminant validity of the scale in this study is good.

### Common Method Bias Test

Common method bias: since the measurement of constructs in this paper uses scales, there may be a problem of common method bias. This study adopted a series of control procedures to reduce the interference caused by common method bias, such as emphasizing the anonymity of this study and improving the items and order of the scale. In this paper, controlling for the effects of an unmeasured latent methods factor (ULMC) was used to test whether there was a common method bias in the study. We build two models by using AMOS 24.0. The model with both method and substantive components could be compared to an identical model except with construct correlations constrained to the values obtained in the substantive-only model. If the two models are significantly different, there is evidence of method bias (Richardson et al., 2009). The comparison results of the main fit indicators of the two models are as follows: ∆RMSEA = 0.007 < 0.05, ∆TLI = 0.01 < 0.1, ∆CFI = 0.01 < 0.1, the above results show that the degree of model fit is not significantly improved after adding the common method factor, so there is no significant common method bias in this study.

## Results

### Main Effect of Positive Emotional Appeal

According to the results of correlation test, consumers’ perceived green value, perceived green responsibility, green purchase behavior, and middle doctrine were highly correlated at the test level of 0.01. Under four different types of positive emotional appeals, the mean comparison of different positive emotions is displayed in figure. As can be seen in figure, compared with the other three emotions, the yearning appeal leads to the highest degree of perceived green value, perceived green responsibility, and green purchase behavior.

**Figure 4:**
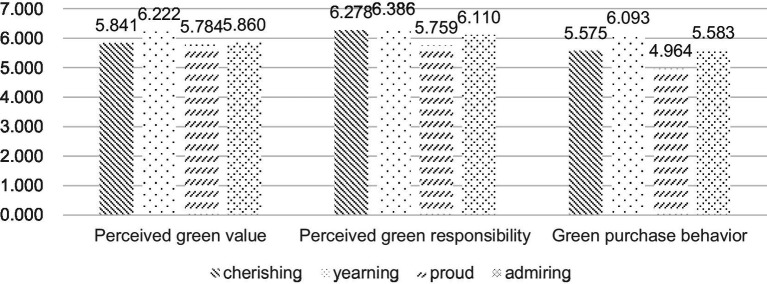
The mean comparison of different positive emotions.

Then we classified four kinds of positive emotional appeals into two specific dimensions, and conducted independent sample T test in different groups, the results of which are shown in table. It can be seen from Table 3 that there is significant difference in the positive effects of the cherishing and yearning appeals on consumers’ perceived green value and green purchase behavior, but there is no significant difference in the positive effects on consumers’ perceived green responsibility, that is, compared with the cherishing appeal, the yearning appeal has a more positive impact on consumers’ perceived green value and green purchase behavior. Table 3 showed that the results of the proud and the admiring group are different to those of the cherishing and yearning group, that is, there is no significant difference in the positive effects of the proud and admiring group on consumers’ perceived green value, but there is significant difference in the positive effects on consumers’ perceived green responsibility and green purchase behavior, that is, compared with the proud appeal, the admiring appeal has a more positive impact on consumers’ perceived green responsibility and green purchase behavior. Therefore, H1 and H3 are accepted and the results in this section partially support H2 and H4 the results of T test is shown in Figure 4.

**Table 3:** Variables’ independent sample *T* test.

Group	Var.	t	DF	Sig.	MD	SD	95% confidence interval	
							The lower limit	The higher limit
1	PGV	−2.509	212	0.013*	−0.381	0.152	−0.680	−0.082
	PGR	−1.023	212	0.307	−0.107	0.105	−0.315	0.0996
	GPB	−3.133	212	0.002**	−0.519	0.166	−0.845	−0.192
2	PGV	−0.456	217	0.649	−0.076	0.167	−0.404	0.252
	PGR	−2.857	217	0.005**	−0.351	0.123	−0.593	−0.109
	GPB	−3.493	217	0.001**	−0.619	0.177	−0.968	−0.270

*PGV = Perceived green value, PGR = Perceived green responsibility, GPB = Green purchase behavior, DF = degrees of freedom, MD = mean difference, and SD=Standard error difference*.

*Group 1: the cherishing and the yearning group. Group 2: the proud and the admiring group*. * *p* < 0.05; ** *p* < 0.01.

### Mediating Effect Test

**Figure 5:**
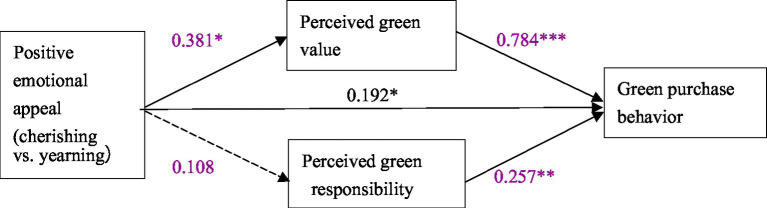
Mediating effect test (cherishing vs. yearning). * *p* < 0.05; ** *p* < 0.01; *** *p* < 0.001.

**Figure 6:**
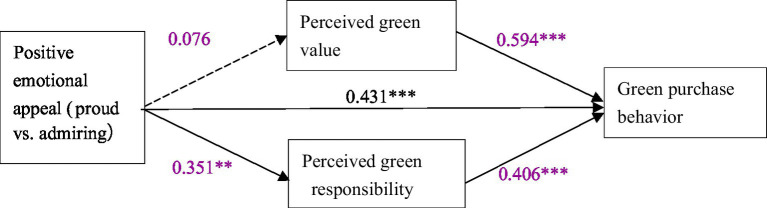
Mediating effect test (proud vs. admiring). ** *p* < 0.01; *** *p* < 0.001.

In order to further test the mediating effect of perceived green value and perceived green responsibility, we adopted the Bootstrap method proposed by Preacher and Hayes (2008), which supports the use of bootstrapping method to handle cases where the probability distribution of the mediating effect value is unknown. The Bootstrapping method does not need to satisfy assumptions such as normal distribution. According to the suggestion of Jie et al. (2012), we adopted the bias-corrected percentile Bootstrapping method and the 95% confidence interval of the mediating effect size was calculated by repeating the sampling process 5,000 times. Adopting PROCESS 3.3 and select model 4 for analysis. Four positive emotions were divided into two groups according to different dimensions to test the mediating effect. The “positive emotional appeal” was transformed into dummy variables. One group took the cherishing appeal as the control, transforming cherishing into 0 and yearning into 1. In the other group, we transform proud into 0 while admiring into 1. Through the analysis with the PROCESS 3.3, the corresponding model path coefficient diagram is shown in Figures 5, 6, and the mediating effect is shown in Table 4.

**Table 4:** Mediating effect of theoretical model.

Explanatory variables	Mediator	Coefficient	S.E.	LLCI	ULCI
Cherishing-Yearning group	The direct effect without the mediator	0.192	0.091	0.014	0.371
	PGV	0.299	0.119	0.071	0.533
	PGR	0.028	0.031	−0.023	0.099
Proud-Admiring group	The direct effect without the mediator	0.431	0.1162	0.202	0.660
	PGV	0.045	0.098	−0.165	0.223
	PGR	0.143	0.058	0.041	0.268

*S.E. = Standard error, LLCI = Lower limit of the confidence interval, ULCI = Upper limit of the confidence interval, PGV = Perceived green value, and PGR = Perceived green responsibility*.

As for the cherishing-yearning group, according to Table 4, the confidence interval of perceived green value does not contain 0, indicating that perceived green value plays a mediating role between positive emotional appeal and green purchase behavior. The confidence interval of perceived green responsibility contains 0, indicating that perceived green responsibility has no significant mediating effect between positive emotional appeal and green purchase behavior. In the absence of mediating variables, the confidence interval of the direct effect of positive emotional appeals on green purchase behavior does not contains 0, indicating that the direct effect is significant. Therefore, perceived green responsibility plays a partial mediating role between positive emotional appeal and green purchase behavior.

By analyzing the proud-admiring group in the same way, it can be found that the mediating effect of perceived green value is not significant, while the mediating effect of perceived green responsibility is significant. In the absence of mediating variables, the confidence interval of the direct effect of positive emotional appeal on green purchase behavior does not include 0, indicating that the direct effect is significant, which proves that perceived green responsibility plays a partial mediating role in the path of positive emotional appeal affecting green purchase behavior.

In conclusion, H2 and H4 is proved to be true.

### Moderating Effect Test of Emotional Arousal

**Figure 7:**
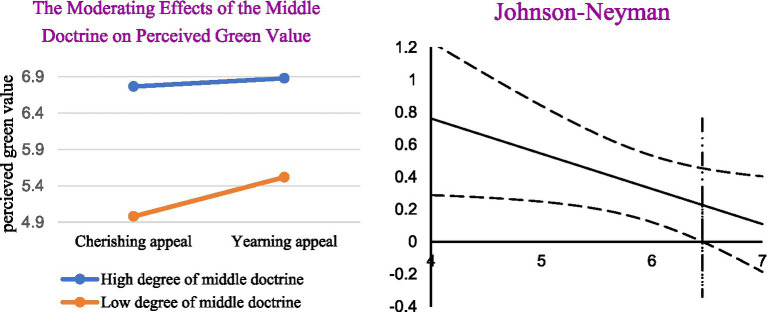
The moderating effects of the middle doctrine on perceived green value and floodlight analysis.

In this section, we test the moderating effect of the middle doctrine on “positive emotional appeals-perceived green value,” “positive emotional appeals-perceived green responsibility,” and “positive emotional appeals-green purchase behavior.” Since the theoretical model of this study is a moderated mediation model, we adopt PROCESS 3.3 of Hayes and select model 8 for analysis. In group 1, the results are displayed in Table 5, which showed that coefficients a31 are significant and the sign of the regression coefficient is negative. It turns out that the middle doctrine significantly negatively moderates the influence of positive emotional appeals on perceived green value. H5a are proved to be true. The corresponding moderating effects of the middle doctrine is show in Figure 7. We probed this interaction with floodlight analysis using the Johnson–Neyman technique to identify the regions of middle doctrine for which the effect of displayed quantity was significant (Spiller et al., 2012). Results revealed that the impact of yearning appeals on consumers’ green purchasing behavior is significant stronger than cherishing appeals’ when the degree of middle doctrine was less than 6.46. The corresponding results of moderating effects of the middle doctrine and floodlight analysis are show in Figure 7. The coefficients a32 and c3′ are not significant. H5b is not accepted. In group 2, the result showed that all the coefficients a31, a32, and c3′ are not significant, indicating that significant moderating effects of the middle doctrine on the excepted paths above do not exist. H5c and H5d are not accepted. In addition, the R2 of each liner regression model is significant, indicating that independent variables selected in this study have a strong explanatory power for the dependent variable.

**Table 5:** Moderating effect test of emotional arousal.

Group	Moderator	Antecedent		PGV				PGR				GPB		
				Coe.	S.E.	P		Coe.	S.E.	P		Coe.	S.E.	P
1	MD	PEA	a_11_	0.327	0.052	**	a_12_	0.071	0.075	0.350	c_1_’	0.209	0.091	*
		PGV	-	-	-	-	-	-	-	-	b_1_	0.734	0.065	***
		PGR	-	-	-	-	-	-	-	-	b_2_	0.202	0.091	*
		MD	a_21_	0.801	0.053	***	a_22_	0.546	0.039	***	c_2_’	0.1174	0.071	0.101
		PEA*MD	a_31_	−0.217	0.108	*	a_32_	−0.031	0.078	0.691	c_3_’	−0.025	0.093	0.786
		R2	0.5473***				0.4940***				0.7293***			
		F	84.6384				68.3525				112.1030			
2	MD	PEA	a_11_	0.031	0.119	0.794	a_12_	0.3221	0.097	**	c_1_’	0.4465	0.115	***
		PGV	-	-	-	-	-	-	-	-	b_1_	0.5021	0.070	***
		PGR	-	-	-	-	-	-	-	-	b_2_	0.3520	0.086	***
		MD	a_21_	0.8151	0.06	***	a_22_	0.5244	0.046	***	c_2_’	0.1933	0.078	*
		PEA*MD	a_31_	0.096	0.112	0.394	a_32_	−0.077	0.091	0.401	c_3_’	0.1094	0.106	0.304
		R2	0.4974***				0.4064***				0.6295***			
		F	70.9188				49.0587				213.0000			

*Coe. = Coefficient, S.E. = Standard error, P = p value, PGV = Perceived green value, PGR = Perceived green responsibility, GPB = Green purchase behavior, PEA = Positive emotional appeal, and MD = Middle doctrine. Group 1: cherishing* vs. *yearning. Group 2: proud* vs. *admiring*. * *p* < 0.05; ** *p* < 0.01; *** *p* < 0.001.

### Discussion

The aim of this study was to explore the influence effect and mechanism of positive emotional appeals on green purchase behavior, enriching the research of positive emotions on green purchase behavior. At present, scholars’ studies have gradually shifted from the cognitive to the emotional level to seek the influencing factors of green purchase behavior; nevertheless, most of which are lack of relevant empirical studies. Based on the results of previous qualitative research and the direction of national policies, it is the first time that creatively introduces the two specific positive emotions into study, including the cherishing and the yearning. At the same time, combined with the Chinese traditional cultural background, this paper incorporated middle doctrine into this theoretical model as a moderating variable, which is helpful to promote the localization of positive emotion and green purchase behavior in the field of consumer behavior.

In this study, we discovered that the diverse types of positive emotional appeals exerted different influence on consumers’ perceived green value, perceived green responsibility, and green purchase behavior. Compared with the cherishing appeal, the yearning appeal is more effective in promoting the perceived green value and green purchase behavior. Compared with the proud appeal, the admiring appeal is more effective in promoting the perceived green responsibility and green purchase behavior. This conclusion confirms Fredrickson's (1998) broaden-and-build model of positive emotion theory. However, it is on the contrary to the results studied by Wang et al. (2017). He found that there is no difference between the proud and the admiring in the green purchase decision-making process.

We found that under the state of positive emotion, perceived green value has significant mediating effect on the relationship between positive emotional appeals (cherishing vs. yearning) and green purchase behaviors. This is consistent with the research conclusions of Shamdasani et al. (1993), Choi and Kim (2005), and Wang et al. (2017). However, perceived green value does not play a mediating role in the path of positive emotional appeals (proud vs. admiring) affecting green purchase behavior. On the contrary, consumers’ perceived green responsibility positively affects their green purchase behavior, and it can significantly mediate the relationship between positive emotional appeals (proud vs. admiring) and green purchase behavior. This finding again validates the responsible environmental behavior model proposed by Hines et al. (1987). Furthermore, this is also consistent with the research conclusions of Scott et al. (2000), Chen (2009), Zhang et al. (2013), and Peng et al. (2014) that the sense of responsibility notably improves green consumption. The sense of responsibility reflects the individual’s internal quality, as the driving factor, affecting the individual’s behavior. The individual internalizes the sense of ecological responsibility in the mind and then obtains the embodiment from the individual’s ecological behavior. Under the stimulation of positive emotional appeal, individuals perceive ecological responsibility and externalize it through ecological behavior. However, perceived green responsibility does not play a mediating role in the path of positive emotional appeals (cherishing vs. yearning) affecting green purchase behavior. The above findings support our previous hypothesis.

Finally, middle doctrine significantly negatively moderates the relationship between positive emotional appeals (cherishing vs. yearning) and perceived green value but no evidence was found to support the moderating effect of middle doctrine on the other paths (positive emotional appeals-perceived green responsibility and positive emotional appeals-green purchase behavior). That is, for consumers with different degrees of middle doctrine, admiring appeal (vs. proud appeals) has a stronger impact on the perceived green responsibility and green purchasing behavior. Our explanation for this result is that consumers with strong moderate values hold such a view that the existence of the individual depends on the relationship of the whole and at the same time has an impact on the whole, so the behavior of others has a stronger impact on them. Furthermore, admiring appeal makes consumers with a higher degree of moderate values pay attention to the green buying behavior of others. Therefore, for consumers with a higher degree of moderate values, the effect of admiring appeal is also higher than that of proud appeal.

## Theoretical Contribution

This research makes three main theoretical contributions: First, our study revealed the different effects of the four dimensions of positive emotional appeal on consumers’ green purchase behavior. Existing research on emotional appeal mainly focuses on the difference in the impact of rational appeal and emotional appeal (Matthes et al., 2014), but there is a lack of in-depth research on different dimensions of positive emotional appeals. Based on four typical positive emotions extracted by Wang (2015) through qualitative research, we further revealed their influence on consumers’ green purchasing behavior through quantitative methods. Our work fills a gap in previous research.

Second, this research advances understanding of how four dimensions of positive emotional appeals shape consumers’ green purchasing behavior. This study verified that cherishing appeal and yearning appeal lead to differences in consumers’ perceived green value, which further affects their green purchase behavior. Positive emotional appeals affect consumers’ perceived value, which is a brand-new point of view. This study also proved that proud appeal and admiring appeal have different effects on consumers’ perceived green responsibility and further influence consumers’ green purchase behavior. The above viewpoints and conclusions provide a new perspective, which is helpful to understand the impact of positive emotional appeals on consumers’ green consumption behavior. At the same time, this paper incorporated middle doctrine into this theoretical model as a moderating variable, which is helpful to promote the localization of positive emotion and green purchase behavior in the field of consumer behavior.

Third, this study expanded the scope of application of the Broaden-and-Build Theory of positive emotions and prospect theory. For the first time, we use prospect theory to explain that consumers’ perceived differences in green value and green responsibility are due to the selection of different reference points (present or future and self or others) under different emotional appeal stimuli. This provides a new perspective for other researchers trying to understand the impact of emotional appeal.

## Policy Implications and Directions for Future Research

The research conclusion provides valuable marketing inspiration and policy management experience for both enterprise managers and government policymakers. Firstly, make full advantage of positive emotional appeal to guide consumers to green purchase sustainably. For enterprise, in the process of promoting the products, it is of great significance to add positive emotional attributes to capture the consumer’s inner demand and arouse their inner resonance, guiding them to buy. At the same time, the government is supposed to attach prime importance to cultivating and enhancing positive emotional attainments and strengthen their recognition of ecological and environmental behaviors, so as to encourage individuals strive for a green ecological environment and actively participate in the sustainable green purchase. Secondly, the cherishing and admiring appeals should be fully valued. To be specific, enterprises can incorporate beautiful ecological environment pictures or videos into the advertisements. In addition, short films, stories, or music can be broadcast to publicize individuals’ green purchase behavior, in order to infect more individuals to join the team for affirming and appreciating others’ environmental protection behavior. Finally, emphasize the role of green responsibility to enhance consumers’ inner awareness of ecological environmental protection. When making product marketing plans, enterprises should not only comprehensively introduce the unique performance of products, but also integrate some elements which can awaken consumers’ awareness of ecological responsibility, urging consumers to be willing to purchase green products.

This work suggests several interesting opportunities for future research. One is to investigate the long-term effects of different types of emotional appeals on consumers’ green purchasing behavior. Our experimental study confirms that emotional appeals have an impact on consumers’ single green purchase behavior but does not take into account consumers’ long-term green purchase behavior. So, researchers can use the Real-time Longitudinal Methods to investigate whether consumers’ behaviors in a certain period of time have continuity and whether its green buying behavior increases or decreases over time. Another opportunity for further research is to more deeply explore the role of positive emotional appeals. For instance, future research can be conducted on the effect of a combination of different positive emotional appeals on consumers (e.g., one group of consumers is stimulated by cherishing and pride appeals, another group is stimulated by yearning and appreciation appeals) and the effects of other types of affective appeals on consumers.

## Data Availability Statement

The original contributions presented in the study are included in the article/Supplementary Material, further inquiries can be directed to the corresponding author.

## Author Contributions

JianmW: conceptualization, data curation, and writing-original draft preparation. ZH: methodology and supervision. JiangW: methodology and software. JB: visualization and investigation. JG: methodology and investigation. XY: conceptualization, methodology, software, and manuscript revision. All authors contributed to the article and approved the submitted version.

## Funding

This study is supported by Major Project of the National Social Science Fund of China (20ZDA087).

## Conflict of Interest

The authors declare that the research was conducted in the absence of any commercial or financial relationships that could be construed as a potential conflict of interest.

## Publisher’s Note

All claims expressed in this article are solely those of the authors and do not necessarily represent those of their affiliated organizations, or those of the publisher, the editors and the reviewers. Any product that may be evaluated in this article, or claim that may be made by its manufacturer, is not guaranteed or endorsed by the publisher.

## Footnotes

^1^International Institute of Green Finance: Analysis of the current situation of green consumption in the field of e-commerce 2021-12-17. Available at: http://iigf.cufe.edu.cn/info/1012/4475.htm

## References

[ref601] Abrahamse W. Steg L. (2009). How do socio-demographic and psychological factors relate to households’ direct and indirect energy use and savings? J. Econ. Psychol. 30, 711–720. doi: 10.1016/j.joep.2009.05.006

[ref1] Algoe Sara B. Jonathan H. (2009). Witnessing excellence in action: the 'other-praising' emotions of elevation, gratitude, and admiration. J. Posit. Psychol. 4, 105–127. doi: 10.1080/17439760802650519, PMID: 19495425 PMC2689844

[ref2] Antonetti P. Maklan S. (2014). Exploring Postconsumption guilt and pride in the context of sustainability. Psychol. Mark. 31, 717–735. doi: 10.1002/mar.20730

[ref3] Bandura A. (2001). Social cognitive theory of mass communication. Media Psychol. 3, 265–299. doi: 10.1207/S1532785XMEP0303_03

[ref4] Berger I. E. Corbin R. M. (1992). Perceived consumer effectiveness and faith in others as moderators of environmentally responsible behaviors. J. Pub. Policy Market 11, 79–89. doi: 10.1177/074391569201100208

[ref5] Chan K. K. W. (1996). Chinese Viewers’ perception of informative and emotional advertising. Int. J. Advert. 15, 152–166. doi: 10.1080/02650487.1996.11104644

[ref6] Chan R. Lau L. (2000). Antecedents of green purchases: a survey in China. J. Consum. Mark. 17, 338–357. doi: 10.1108/07363760010335358

[ref7] Chen L. H. (2009). Research on energy consumption behavior of urban residents. Doctoral Dissertation of Dalian University of Technology.

[ref910] Chen K. Deng T. (2017). Research on environmental attitudes, guide information and green commuting intentions. J. Arid Land Resour. Environ. 31, 191–196. doi: 10.13448/j.cnki.jalre.2017.099

[ref602] Choi S. M. Kim Y. (2005). Antecedents of green purchase behavior: An examination of collectivism, environmental concern, and pce. Adv. Consumer Rese. Assoc. Consumer Res. 32, 592–599.

[ref603] Darley W. K. Smith R. E. (1993). Advertising claim objectivity: Antecedents and effects. J. Mark. 57, 100–113. doi: 10.1177/002224379102800305

[ref604] Dodds W. B. Monroe K. B. Grewal D. (1991). Effects of price, brand, and store information on buyers’ product evaluations. J. Mark. Res. 28, 307–319. doi: 10.1177/002224379102800305

[ref605] Du J. Ran M. Cao P. (2014). Context-contingent effect of zhongyong on employee innovation behavior. Acta Psychol. Sin. 46, 113–124. doi: 10.3724/SP.J.1041.2014.00113

[ref9] Feng X. Zhang X. (2007). Self-conscious emotions: Advanced emotions of human. Adv. Psychol. Sci. 15, 878–884. doi: 10.3969/j.issn.1671-3710.2007.06.003

[ref10] Fredrickson B. L. (1998). What good are positive emotions. Rev. Gen. Psychol. 2, 300–319. doi: 10.1037/1089-2680.2.3.300, PMID: 21850154 PMC3156001

[ref12] Han H. L. Zou T. Q. Zhuang F. P. (2017). Research on function paths of corporate brand image on purchase intention: based on China's multi-national enterprises. J. Central University Finance Economics 8, 91–99.

[ref606] Harth N. S. Leach C. W. Kessler T. (2013). Guilt, anger, and pride about in-group environmental behaviour: Different emotions predict distinct intentions. J. Environ. Psychol. 34, 18–26. doi: 10.1016/j.jenvp.2012.12.005

[ref13] Helen M. Immordino-Yang Sylvan L. (2010). Admiration for virtue: neuroscientific perspectives on a motivating emotion. Contemp. Educ. Psychol. 35, 110–115. doi: 10.1016/j.cedpsych.2010.03.003

[ref14] Hines J. M. Hungerford H. R. Tomera A. N. (1987). Analysis and synthesis of research on responsible environmental behavior: a meta-analysis. J. Environ. Educ. 18, 1–8. doi: 10.1080/00958964.1987.9943482

[ref18] Jie F. Min-qiang Z. Haw-jeng C. (2012). Mediation analysis and effect size measurement: Retrospect and Prospect. Psychol. Dev. Educ. 28, 105–111. doi: 10.16187/j.cnki.issn1001-4918.2012.01.015

[ref19] Kanchanapibul M. Lacka E. Wang X. J. Chan H. K. (2014). An empirical investigation of green purchase behaviour Among the young generation. J. Clean. Prod. 66, 528–536. doi: 10.1016/j.jclepro.2013.10.062

[ref20] Keltner D. Haidt J. (1999). Social functions of emotions at four levels of analysis. Cognit. Emot. 13, 505–521. doi: 10.1080/026999399379168

[ref21] Kollmuss A. Agyeman J. (2002). Mind the gap: why do people act environmentally and what are the barriers to pro-environmental behavior. Environ. Educ. Res. 8, 239–260. doi: 10.1080/13504620220145401

[ref22] Kronrod A. Grinstein A. Wathieu L. (2012). Go green! Should environmental messages be so assertive? J. Mark. 76, 95–102. doi: 10.1509/jm.10.0416

[ref607] Ku H. H. Kuo C. C. Wu C. L. Wu C. Y. (2012). Communicating green marketing appeals effectively. J. Advert. 41, 41–50. doi: 10.1080/00913367.2012.10672456

[ref24] Lao K. F. (2013). Research on the influence mechanism of Consumer’s innovation on green consumption behavior. *Nankai*. Manag. Rev. 16, 113–132.

[ref25] Larrick R. P. Heath C. Wu G. (2009). Goal-induced risk taking in negotiation and decision making. Soc. Cogn. 27, 342–364. doi: 10.1521/soco.2009.27.3.342

[ref26] Lei S. U. N. Kunhao C. A. I. (2016). A study on the effect of deceptive environmental claims in Greenwash advertising. Chinese J. Journal. Commun 38, 134–151. doi: 10.13495/j.cnki.cjjc.2016.12.008

[ref28] Liu J. R. (2015). Analysis on establishing positive learning model. Edu. Asia Pacific 1, 129–137. doi: 10.16550/j.cnki.2095-9214.2015.01.026

[ref29] Liu Z. S. Gu D. Jiang J. (2020). Gain or loss? The influence of advertising target framework on green consumption intention. Chin. J. Clin. Psych. 28, 99–104. doi: 10.16128/j.cnki.1005-3611.2020.01.022

[ref30] Maloney J. Lee M. Y. Jackson V. Kimberly A. M.-S. (2014). Consumer willingness to purchase organic products: application of the theory of planned behavior. J. Glob. Fash. Market. 5, 308–321. doi: 10.1080/20932685.2014.925327

[ref31] Matsueda R. L. Akers R. L. (1999). Social learning and social structure: a general theory of crime and deviance. Contemp. Sociol. 28:100. doi: 10.2307/2653905

[ref32] Matthes J. Wonneberger A. Schmuck D. (2014). Consumers’ green involvement and the persuasive effects of emotional versus functional ads. J. Bus. Res. 67, 1885–1893. doi: 10.1016/j.jbusres.2013.11.054

[ref34] Meneses G. D. (2010). Refuting fear in heuristics and in recycling promotion. J. Bus. Res. 63, 104–110. doi: 10.1016/j.jbusres.2009.02.002

[ref35] Onwezen M. C. Antonides G. Bartels J. (2013). The norm activation model: an exploration of the functions of anticipated pride and guilt in pro-environmental behaviour. J. Econ. Psychol. 39, 141–153. doi: 10.1016/j.joep.2013.07.005

[ref36] Onwezen M. C. Bartels J. Antonides G. (2014). Environmentally friendly consumer choices: cultural differences in the self-regulatory function of anticipated pride and guilt. J. Environ. Psychol. 40, 239–248. doi: 10.1016/j.jenvp.2014.07.003

[ref37] Pan Y. Gao L. Wang F. H. (2009). Influence of lifestyle and customer perceived value on Chinese Consumers’ Purchasie behavior. J. Syst. Manag. 18, 601–607.

[ref38] Pan Y. Gao L. Zhang X. Wan Y. (2014). Research on consumer values in the context of Chinese Culture–Scale development and comparison. Manage. World 20, 90–106. doi: 10.19744/j.cnki.11-1235/f.2014.04.010

[ref39] Peattie K. (2010). Green consumption: behavior and norms. Annu. Rev. Environ. Resour. 35, 195–228. doi: 10.1146/annurev-environ-032609-094328

[ref40] Peng D. Y. Ma S. Y. Bai R. (2014). Empirical analysis on influencing factors of low-carbon consumption behavior of urban residents–A case study of Nanchang city. J. Ecol. Eco 30, 119–122. doi: 10.3969/j.issn.1009-6094.2011.05.030

[ref608] Preacher K. J. Hayes A. F. (2008). Asymptotic and resampling strategies for assessing and comparing indirect effects in multiple mediator models. Behav. Res. Methods 40, 879–891. doi: 10.3758/BRM.40.3.879 18697684

[ref41] Qiu L. Zheng X. Wang Y. F. (2008). Revision of PANAS. Appl. Psychol. 23, 249–254.

[ref42] Rajesh K. C. Gerard J. T. Deborah J. M. Pattana T. (2001). What to say when: advertising appeals in evolving markets. J. Mark. Res. 38, 399–414. doi: 10.1509/jmkr.38.4.399.18908

[ref609] Ren J. (2015). Research on the Effects of Reference Points on Online Consumers’ Puchasing Decisions. Doctoral Dissertation of China Agricultural University.

[ref43] Richardson H. A. Simmering M. J. Sturman M. C. (2009). A tale of three perspectives: examining post hoc statistical techniques for detection and correction of common method variance. Organ. Res. Methods 12, 762–800. doi: 10.1177/1094428109332834

[ref44] Sarapin S. H.. (2015). Identifying admired models to increase emulation: development of a multidimensional admiration scale. Measurement and Evaluation in Counseling and Development: The Official Publication of the Association for Assessment in Counseling and Education, a Division of the American Counseling Association 48, 95–108.

[ref46] Schuhwerk M. E. Lefkoff-Hagius R. (1995). Green or non-green: does type of appeal matter when advertising a green product. J. Advert. 24, 45–54. doi: 10.1080/00913367.1995.10673475

[ref47] Scott D. Parker P. Rowlands I. H. (2000). Determinants of energy efficiency choices in the home: a case study of Waterloo region. Environments 28, 75–100.

[ref611] Shamdasani P. Chon-Lin G. Richmond D. (1993). Exploring green consumers in an oriental culture: Role of personal and marketing mix factors. Auk 117, 936–942.

[ref48] Shen Z. F. Yang X. M. Zhao D. C. (2011). Development of quality of gratitude questionnaire for college students. *Chinese* J. Clin. Psychol. 19, 35–37.

[ref49] Spiller S. A. Fitzsimons G. J. Lynch J. G. Jr. Mcclelland G. H. (2012). Spotlights, floodlights, and the magic number zero: simple effects tests in moderated regressiohn. J. Mark. Res. 50, 277–288. doi: 10.1509/jmr.12.0420

[ref51] Tanner C. Kast S. W. (2003). Promoting sustainable consumption: determinants of green purchases by Swiss consumers. Psychol. Mark. 20, 883–902. doi: 10.1002/mar.10101

[ref52] Thøgersen J. Ölander F. (2002). Human values and the emergence of a sustainable consumption pattern: a panel study. J. Econ. Psychol. 23, 605–630. doi: 10.1016/S0167-4870(02)00120-4

[ref54] Tversky K. A. (1979). Prospect theory: an analysis of decision under risk. Econometrica 47, 263–291. doi: 10.2307/1914185

[ref612] Venkatesh V. Morris M. G. Davis G. B. Davis F. D. (2003). User acceptance of information technology: Toward a unified view. MIS Quarterly 27, 425–478. doi: 10.2307/30036540

[ref55] Wallace S. G. Jordan E. (2017). How goal specificity shapes motivation: A reference points perspective. J. Consum. Res. 44, 1033–1051. doi: 10.1093/jcr/ucx082

[ref56] Wang J. M. (2015). Environmental emotion dimension structure and its influence on carbon consumption and emission reduction behavior–two factor theory hypothesis of emotion and behavior and its verification. Manage. World 32, 82–95. doi: 10.19744/j.cnki.11-1235/f.2015.12.008

[ref613] Wang J. M. Wang C. C. Wu L. C. (2017). The influence mechanism of green emotional appeal on the decision-making process of green purchase. Manag. Sci. 30, 38–56. doi: 10.3969/j.issn.1672-0334.2017.05.004

[ref61] Wang J. M. Wu L. C. (2015). Emotion-behavior two factor model for green purchasing: Hypotheses and tests. Manag. Sci. 28, 80–94. doi: 10.3969/j.issn.1672-0334.2015.06.007

[ref63] Wang J. M. Zheng R. R. (2011). Mechanism of influence of psychological awareness factors on ecological civilization behavior of consumers. J. Manag. 8, 1027–1035. doi: 10.3969/j.issn.1672-884X.2011.07.012

[ref64] Wareham J. Boots D. P. Chavez J. M. (2009). A test of social learning and intergenerational transmission among batterers. J. Crim. Just. 37, 163–173. doi: 10.1016/j.jcrimjus.2009.02.011

[ref65] Watson D. Tellegen A. (1985). Toward a consensual structure of mood. Psychol. Bull. 98, 219–235. doi: 10.1037/0033-2909.98.2.219, PMID: 3901060

[ref66] Watson D. Wiese D. Vaidya J. Tellegen A. (1999). The two general activation Systems of Affect: structural findings, evolutionary considerations, and psychobiological evidence. J. Pers. Soc. Psychol. 76, 820–838. doi: 10.1037/0022-3514.76.5.820

[ref67] Williams L. A. DeSteno D. (2009). Pride: adaptive social emotion or seventh sin? Psychol. Sci. 20, 284–288. doi: 10.1111/j.1467-9280.2009.02292.x 19207690

[ref68] Xu X. Arpan L. M. Chen C. F. (2015). The moderating role of individual differences in responses to benefit and temporal framing of messages promoting residential energy saving. J. Environ. Psychol. 44, 95–108. doi: 10.1016/j.jenvp.2015.09.004

[ref69] Yang D. Lu Y. Zhu W. Su C. (2015). Going green: how different advertising appeals impact green consumption behavior. J. Bus. Res. 68, 2663–2675. doi: 10.1016/j.jbusres.2015.04.004

[ref614] Yang Z. F. (2009). A case of attempt to combine the chinese traditional culture with the social science: The social psychological research of “zhongyong”. RUC 23, 53–60.

[ref70] Zagzebski L. (2015). I-admiration and the admirable. Aristotelian Soc. Suppl. 89, 205–221. doi: 10.1111/j.1467-8349.2015.00250.x

[ref72] Zhang J. Xu P. P. Sang X. Y. (2013). Research on green consumption behavior under the guidance of social responsibility. China Securities Futures 2, 198–199.

[ref73] Zhi Y. Zhao Q. Wang J. (2017). Influence of ad appeal and environmental attitudes on the psychological effect of green products ads. Econ. Manag. 31, 65–71. doi: 10.3969/j.issn.1003-3890.2017.01.012

